# A Bayesian Analysis of Plant DNA Length Distribution via *κ*-Statistics

**DOI:** 10.3390/e24091225

**Published:** 2022-09-01

**Authors:** Maxsuel M. F. de Lima, Dory H. A. L. Anselmo, Raimundo Silva, Glauber H. S. Nunes, Umberto L. Fulco, Manoel S. Vasconcelos, Vamberto D. Mello

**Affiliations:** 1 Departamento de Física, Universidade do Estado do Rio Grande do Norte, Natal 59072-970, RN, Brazil; 2 Departamento de Física, Universidade Federal do Rio Grande do Norte, Natal 59072-970, RN, Brazil; 3 Departamento de Ciências Vegetais, Universidade Federal Rural do Semi-Árido, Mossoró 59625-900, RN, Brazil; 4 Departamento de Biofísica e Farmacologia, Universidade Federal do Rio Grande do Norte, Natal 59072-970, RN, Brazil

**Keywords:** DNA, *cucurbitaceae*, non-additive statistics

## Abstract

We report an analysis of the distribution of lengths of plant DNA (exons). Three species of *Cucurbitaceae* were investigated. In our study, we used two distinct κ distribution functions, namely, κ-Maxwellian and double-κ, to fit the length distributions. To determine which distribution has the best fitting, we made a Bayesian analysis of the models. Furthermore, we filtered the data, removing outliers, through a box plot analysis. Our findings show that the sum of κ-exponentials is the most appropriate to adjust the distribution curves and that the values of the κ parameter do not undergo considerable changes after filtering. Furthermore, for the analyzed species, there is a tendency for the κ parameter to lay within the interval (0.27;0.43).

## 1. Introduction

There are 15 tribes in the family Cucurbitaceae [1]. The tribe Cucurbitae, which has an almost completely American distribution, consists of 11 genera, including the genus *Cucurbita*. The genus *Cucurbita* (Cucurbitaceae) has five major domesticated species: *Cucurbita moschata*, *Curcurbita pepo*, *Cucurbita maxima*, *Cucurbita argyrosperma*, and *Cucurbita ficifolia* [2,3].

The first three species cited are the most economically important as a popular food resource [4]. The fruits of the species are incredibly diverse, differing greatly in shape, surface topography, color, size, and color pattern [5]. Among them, *C. pepo* is the genus’ most phenotypically variable species and has eight cultivar groups with edible fruits (groups) [6]. The second most diversified species in the genus is thought to be *C. moschata* [7].

All Cucurbita species have 20 pairs of chromosomes (2n=2x=40), making them all diploid. The theory that Cucurbiteae underwent one whole-genome duplication as a result of their high chromosome number has gained traction [8,9]. The tribe Cucurbiteae plant species, including the zucchini (*C. pepo*), pumpkin (*C. moschata* and *C. maxima*), and silver-seed gourd (*C. argyrosperma*), all suffered whole-genome duplication events, according to a number of studies [9,10,11].

There are few estimates of genome size in the genus *Cucurbita*. However, studies have shown relatively small genome sizes. The genome sizes of *C. maxima* and *C. moschata* were estimated to be 271.40 and 269.90Mb, respectively, [9], while the genome size in *C. pepo* was estimated to be 263.0Mb [10]. Concerning the number of genes, the estimated values for *C. maxima*, *C. moschata*, and *C. pepo* were 32.076;32.205 [9]; and 27.868 genes [10], respectively.

On the other hand, numerous models based on statistical physics consistently attempt to represent statistical features, such as long-range and short-range correlations, in light of the large DNA sequence data. Some approaches used statistical tools in connection with random-walk simulations [12,13,14], wavelet transforms [15,16], 1D Ising models [17] (see e.g., [18] and references therein), and Tsallis’ statistics together with Machine Learning [19]. Many live creatures’ coding and non-coding sequence length distributions have been studied by some models in relation to long- and short-range correlations [20,21,22,23]. Non-additive entropy-based statistical physics methods have recently been actively advocated for use in complex system research [24,25]. In this case, the Kaniadakis entropy yields a power-law distribution rather than an exponential one and depends on a free parameter (the κ parameter) [26,27,28]. The κ-statistics arose as a useful statistical tool for many systems (see [29] and references therein). For problems associated with human DNA, see e.g., [30,31].

Additionally, the Bayesian inference has been effectively applied as a useful tool to investigate a number of issues in physics [32] and biophysics [33]. Which DNA models should be valid from the perspective of Bayesian inference is an intriguing subject. Additionally, the challenge in the context of this work would be to investigate an expansion of a model from Ref. [31], but this time in the context of other living structures, such as vegetables.

More recently in [34], statistical models of the Tsallis type provided the distribution of nucleotide chain lengths, successfully capturing the statistical correlations between the parts of the plant (for both coding and non-coding) DNA strands for two species of the Cucurbitaceae family. We expand the paradigm proposed in [31] in the context of vegetables in this article. We especially evaluate the distribution of nucleotide chain lengths measured in base pairs for *Cucurbita maxima*, *Cucurbita moschata*, and *Cucurbita pepo* utilizing κ-deformed statistics in light of the social and economic significance of cucurbits. The most practical model is then chosen using a Bayesian statistical analysis based on the κ-distributions. To the best of our knowledge, this is the first time the size distribution of plant DNA has been realized using a κ-statistical analysis.

## 2. Materials and Methods

We use the κ-statistics, developed by Kaniadakis [26,27,28], to analyze the correlations between the DNA length distributions of some species of the *Cucurbitaceae* family. There are some works in this direction using the Tsallis *q*-statistics [34,35,36]. The κ-entropy and power-law distribution functions naturally arise from the kinetic foundations of κ-statistics. Formally, the κ-framework is based on the κ-exponential and κ-logarithm functions (see Ref. [26]), defined as

(1) $$\begin{array}{ccc} \exp_{\kappa }\left(x\right) & = & {\left[\sqrt{1+\kappa^{2}x^{2}}+\kappa x\right]}^{\frac{1}{\kappa }} \end{array}$$

(2) $$\begin{array}{ccc} \ln_{\kappa }\left(x\right) & = & \frac{x^{\kappa }-x^{-\kappa }}{2\kappa }. \end{array}$$

The parameter κ is restricted to values belonging to the range |κ|<1; for κ=0, these expressions reduce to the usual exponential and logarithmic functions. From the optimization of entropy $S_{\kappa }$ (see Ref. [37]),we can obtain the probability distributions ($P_{\kappa ,1}\left(l\right)$) associated with the quantities of base pairs (bp) for each of the chromosomes of *Cucurbita maxima*, *Cucurbita moschata*, and *Cucurbita pepo*. Mathematically, the Kaniadakis entropy $S_{\kappa }$ is given by

(3) $$S_{\kappa }\left(l\right)=-\frac{1}{2\kappa }\int_{\kappa }\left[\frac{1}{1+\kappa }P_{\kappa }{\left(l\right)}^{\left(1+\kappa \right)}-\frac{1}{1-\kappa }P_{\kappa }{\left(l\right)}^{\left(1-\kappa \right)}\right]dl.$$

The optimization process is well described in Refs. [26,37,38,39,40,41] and gives us $P_{\kappa ,1}\left(l\right)$

(4) $$P_{\kappa ,1}\left(l\right)=\left(1-\kappa^{2}\right)\beta \exp_{\kappa }\left[-\beta l\right].$$

Rewriting (4) with the explicit form of $\exp_{\kappa }\left(-\beta l\right)$ given by (1), and using constraints as in Ref. [41], we get

(5) $$P_{\kappa ,1}\left(l\right)=\frac{\left(1-\kappa^{2}\right)}{L_{\kappa }}{\left[\sqrt{1+\kappa^{2}{\left(-\frac{l}{L_{\kappa }}\right)}^{2}}+\kappa \left(-\frac{l}{L_{\kappa }}\right)\right]}^{\frac{1}{\kappa }}.$$

Here, $L_{\kappa }$ is an adjustable parameter that is related to the mean value of the length distribution, κ is the model’s free parameter which measures the interaction between the nucleotides in the sample, and *l* is the chain of nucleotides’ length, expressed in number of base pairs.

We employ the cumulative probability distribution because the probabilities for lengthy lengths l of the nucleotide chain are subject to significant fluctuations.

We employ the cumulative probability distribution because the probabilities for lengthy lengths l of the nucleotide chain are subject to significant fluctuations, (5) can be found by solving $\Phi \left(l\right)=p\left(l^{\prime }<l\right)=\int_{0}^{l}p\left(l^{\prime }\right)dl^{\prime }$, which provides

(6) $$\begin{array}{c} \Phi_{\kappa ,1}\left(l\right)=1-\frac{1}{2}\left[G_{\kappa }^{+}\left(l\right)+G_{\kappa }^{-}\left(l\right)\right], \end{array}$$

where

(7) $$\begin{array}{c} G_{\kappa }^{\pm }\left(l\right)=\left(1\pm \kappa \right)\exp_{\kappa }^{1\mp \kappa }\left(-\frac{l}{L_{\kappa }}\right). \end{array}$$

Here, Φ(l) denotes the probability of finding the sizes of the bases between 0 and *l*. In Ref. [34], it was proposed a comparison between the *q*-exponential and a sum of *q*-exponentials to explain the DNA length distribution of two species of cucurbits, *Cucumis melo* and *Cucumis sativus*. Based on this work, we propose an analysis of the same type but using the κ-statistics. We assume that the sum of Kaniadakis-type generalized probabilities (already normalized) is given by

(8) $$P_{\kappa ,2}\left(l\right)=\left(1-\kappa^{2}\right)\left[\frac{\gamma_{1}\gamma_{2}}{\gamma_{1}+\gamma_{2}}\right]\left[\exp_{\kappa }\left(-\gamma_{1}l\right)+\exp_{\kappa }\left(-\gamma_{2}l\right)\right],$$

where κ, $\gamma_{1}$, and $\gamma_{2}$ are adjustable parameters and *l* is the length of the nucleotides, respectively. By employing the identical steps as those leading to (6), the cumulative probability distribution is found to be

(9) $$\begin{array}{c} \Phi_{\kappa ,2}\left(l\right)=1-\left[\frac{1}{\gamma_{1}}F_{1,\kappa }\left(l\right)+\frac{1}{\gamma_{2}}F_{2,\kappa }\left(l\right)\right], \end{array}$$

where

(10) $$F_{j,\kappa }\left(l\right)=\frac{\gamma_{1}\gamma_{2}}{\gamma_{1}+\gamma_{2}}\left[\frac{\exp_{\kappa }^{1-\kappa }\left(-\gamma_{j}l\right)}{1-\kappa }+\frac{\exp_{\kappa }^{1+\kappa }\left(-\gamma_{j}l\right)}{1+\kappa }\right],\;\;j=1,2.$$

Initial analyses indicate that, as occurred for the Tsallis’ *q*-statistics [34], the κ-exponential sum model best fits the DNA length distributions of the species studied here. Therefore, we chose to make a comparison between the sum of κ-exponentials (9) and the κ-Maxwellian model (11) below, proposed in [31] to explain the length distribution of human DNA.

(11) $$\Phi_{\kappa ,3}\left(l\right)=1-\exp_{\kappa }\left(-\frac{l^{2}}{\sigma_{\kappa }^{2}}\right)\left[\sqrt{1+\kappa^{2}\frac{l^{4}}{\sigma_{\kappa }^{4}}}+\kappa^{2}\frac{l^{2}}{\sigma_{\kappa }^{2}}\right].$$

The best model to describe the length distributions of the nucleotides for three species of the *Cucurbitaceae* family is obtained by comparing, via Bayesian analysis, the distributions $\Phi_{\kappa ,2}\left(l\right)$ and $\Phi_{\kappa ,3}\left(l\right)$, which are represented by Equations (9) and (11), respectively.

## 3. Results

We use the public database of the National Center for Biotechnology Information (NCBI) [42] and the Comparative Genomics (CoGe) [43]. They are databases that give users access to genetic and biological data. In our analysis, we considered only the coding bases (exons). We define a nucleotide sequence’s length in terms of the l(bp) base pairs. All graphical and data modeling was written in R, a free statistical software [44].

**Figure 1:**
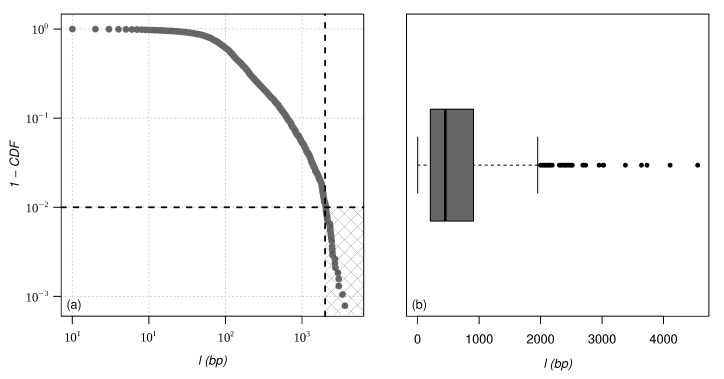
(**a**) Cumulative probability distribution function (CDF) and (**b**) box-plot for chromosome 02 of the species *Cucurbita maxima*. A similar analysis was performed for all chromosomes of the three species of *cucurbitaciae* studied in this paper.

By plotting the cumulative probability distribution function (CDF) and a box plot for chromosome 02 of one of the species studied here (Figure 1), we can see that some points are very far from the distribution and can be considered outliers. There are various techniques for defining, spotting, and dealing with outliers [45]. In this work, we decided to use the box plot approach. Outliers in this approach are points that are below the region Q1−1.5×IQR and above Q3+1.5×IQR, where Q1, Q2, and Q3 are first, second, and third quartile, respectively, and IQR is the interquartile region defined as IQR=Q3−Q1. To prevent these points from influencing the behavior of the proposed models, we decided to remove them. The cut was made around 1% of the cumulative distribution, designated by the hatched square in the lower right corner of Figure 1a. A similar approach has been proposed in [46] to analyze the length distribution of human DNA. Table A1 describes the statistical characteristics of some chromosomes of the three species of *Cucurbitaceae* after removing these outliers.

We decided to analyze the impact this action had on the value of κ, taking into account the cumulative distribution functions (9) and (11). In Table A2 and Table A3, we have the number of nucleotides (N) and the best fit values per κ. The subscripts 0 and *f* represent the values before and after the outliers are removed, and (RD) represents the relative difference between them. The values of RD are smaller than the errors associated with the values of κ in Table A4, Table A5 and Table A6. This work deals with a statistical analysis of the distribution of DNA lengths in plants. Possible biological effects caused by removing nucleotides with large amounts of base pairs were not taken into account.

**Figure 2:**
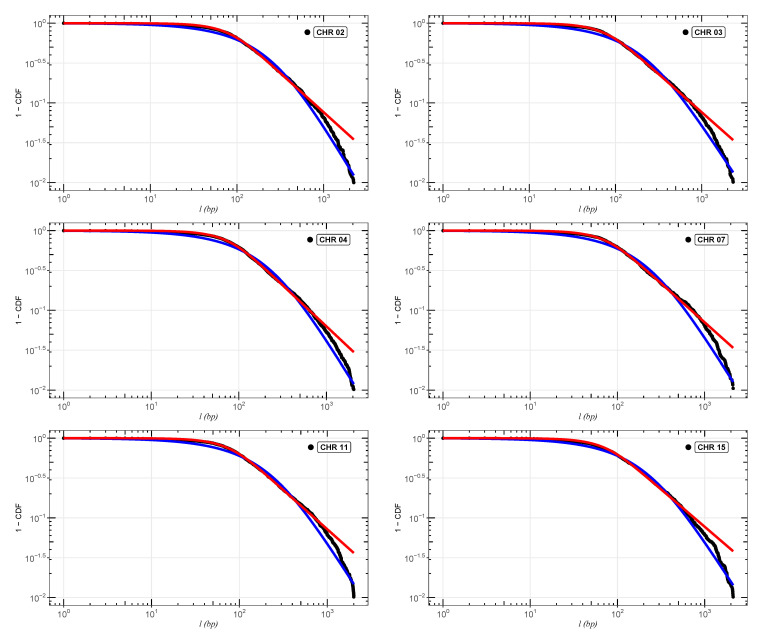
Best fit analysis for the exons of *Cucurbita maxima*. We can observe the adjustments for chromosomes (CHR)02,03,04,07,11, and 15. The blue and red curves are, respectively, the distributions (9) and (11). The other chromosomes follow the same pattern.

**Figure 3:**
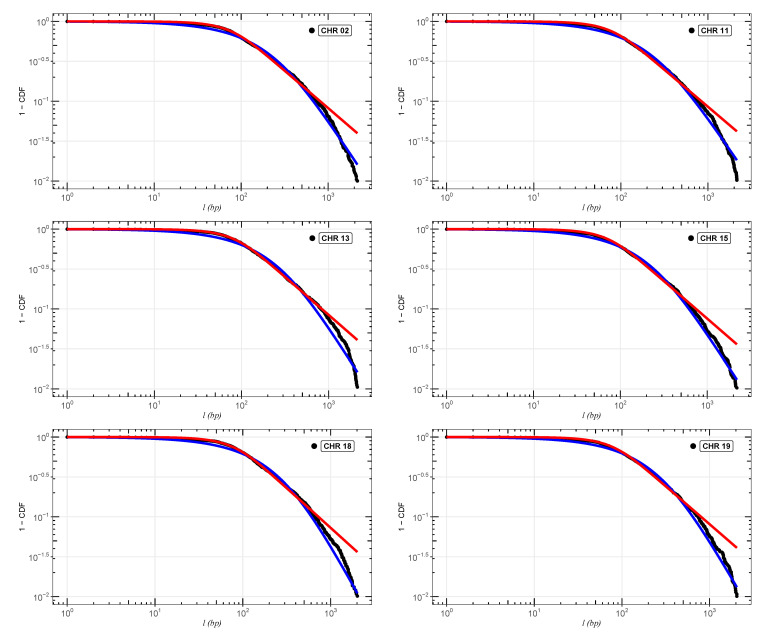
Best fit analysis for the exons of *Cucurbita moschata*. We can observe the adjustments for chromosomes (CHR)02,11,13,15,18, and 19. The blue and red curves are, respectively, the distributions (9) and (11). The other chromosomes follow the same pattern.

**Figure 4:**
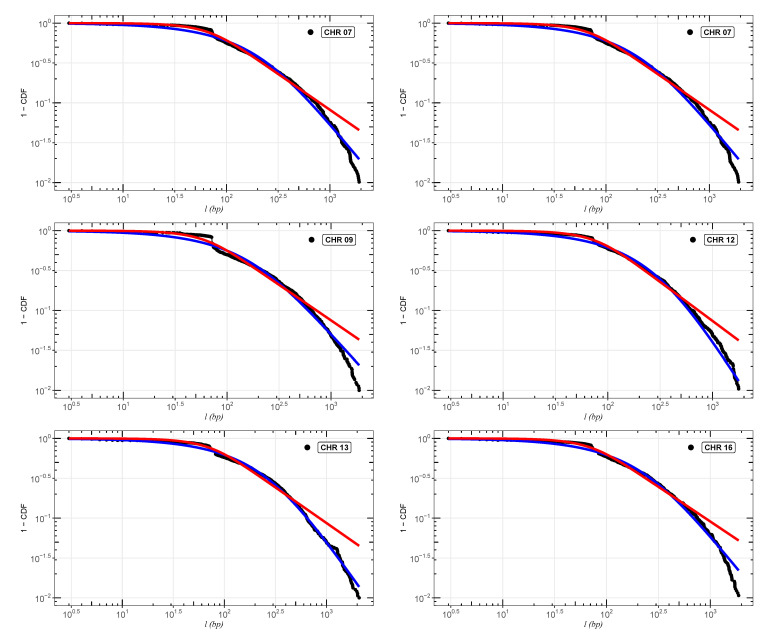
Best fit analysis for the exons of *Cucurbita pepo*. We can observe the adjustments for chromosomes (CHR) 01, 07, 09, 12, 13, and 16. The blue and red curves are, respectively, the distributions (9) and (11). The other chromosomes follow the same pattern.

In Figure 2, Figure 3 and Figure 4, we show the cumulative distributions, for exons, for some chromosomes of *Cucubita maxima*, *Cucurbita moschata*, and *Cucubita pepo*, with the other chromosomes behaving similarly. To get the best fit values for κ, the distribution functions (9) and (11) were fitted to the lengths (*l*). Table A4, Table A5 and Table A6 show all numerical results for the parameters κ, $\gamma_{1}$ and $\gamma_{2}$ for distribution (9) in addition to κ and $\sigma_{\kappa }$ for distribution (11). Chromosome numbers are displayed in the first column (CHR), and the number of nucleotide chains is displayed in the second column (N) (exons). The correlations between the values of *l* are measured by the values of κ [26,27,28,39]. According to [36,47], the coding part of human DNA tends to present short-range correlations. The same behavior for plant DNA can be observed in [34]. This implies κ values close to zero. It is worth remembering that in the limit κ→0, we return to the well-known Boltzmann–Gibbs–Shannon statistics [26].

The models that fit the length distribution Φ(l) the best are determined via Bayesian statistics. By taking into account the probability distribution of the hypotheses, conditioned on the evidence, Bayesian inference describes the relationship between the model and the data, and enables a rational and effective selection of one or more hypotheses [48]. The Bayes’ theorem,

(12) $$P\left(\Phi |D,M\right)=\frac{L(D|\Phi ,M)\cdot P(\Phi |M)}{E(D|M)},$$

offers us the likelihood that, given the data *D*, a posterior model Φ will be correct. For this, the probability of the prior model P(Φ|M) is multiplied by the likelihood function L(D|Φ,M) and divided by the Bayesian evidence E(D|M). Here, we assume the pattern $\chi^{2}={\left(P\left(l^{obs}\right)-P\left(l^{the}\right)\right)}^{2}/\sigma_{obs}^{2}$ for the likelihood function, where $P(l^{obs})$, $P(l^{the})$ and $\sigma_{obs}$ are the cumulative probabilities associated with the observed and the theoretical nucleotide lengths, and observed errors, respectively.

The input parameters used in the prior uniform distribution were obtained from the best fit found by the R-code. This approach, which defines the model parameters’ potential range and significantly affects the Bayesian evidence, is a crucial phase in the study. This condition ensures that the parameters will fall inside the previously identified optimal adjustment range.

In Table A4, we have the parameter ranges for *Cucubita maxima*. Considering all chromosomes (CHR), $\kappa_{M}∼U\left(0.64,0.69\right)$, $\sigma_{\kappa }∼U\left(91,105\right)$, for cumulative distribution (11), and $\kappa_{S}∼U\left(0.24,0.39\right)$, $\gamma_{1}∼U\left(0.0041,0.0087\right)$ and $\gamma_{2}∼U\left(0.0045,0.0088\right)$ for cumulative distribution (9). The process is repeated for the species *Cucubita moschata* in Table A5 and *Cucubita pepo* in Table A6. The MULTINEST algorithm, a Bayesian inference tool that computes the evidence E(D|M) with an associated error estimate, is thus put into practice for each species and each model. It generates posterior samples from distributions that can contain multiple modes and pronounced degeneracy (curves) in high dimensions. More details can be seen in [49,50,51,52,53].

In order to compare the models, we make use of the Bayes factor, which is given by

(13) $$B_{ij}=\frac{E_{i}}{E_{j}}.$$

Here, $E_{j}$ is the evidence of the base model, which is used as a reference. In our case, this is the distribution (9), and $E_{i}$ is the evidence of the model we want to compare, given by distribution (11). We employ the Bayes factor interpretation provided by Jeffrey’s theory [35,54,55,56] to measure whether a model has favorable evidence in comparison to the base model. Table A7 contains the findings for each chromosome.

**Figure 5:**
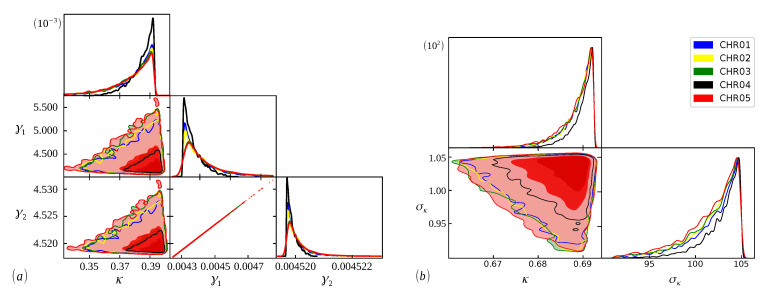
Bayesian analysis for the (9) (**a**) and (11) (**b**) distributions, using chromosomes 01,02,03,04, and 05 of the coding part of *Cucurbita maxima* DNA. The rest of the sample follows a similar pattern.

**Figure 6:**
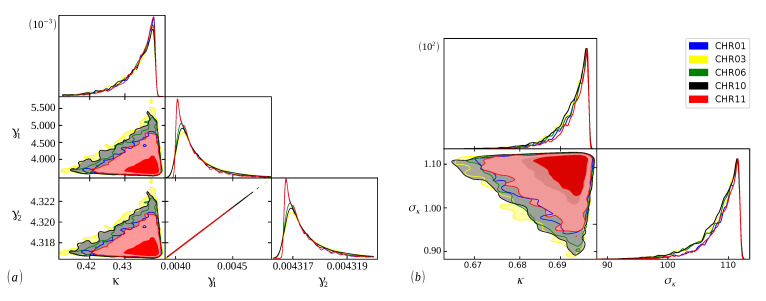
The same as Figure 5, but for chromosomes 01,03,06,10, and 11 of the *Cucurbita moschata* species.

**Figure 7:**
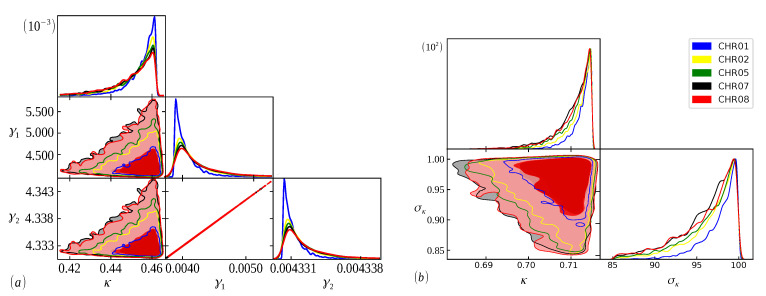
The same as Figure 5, but for chromosomes 01,02,05,07 and 08 of the *Cucurbita pepo* species.

The Bayesian analysis is performed from each model’s range of definite parameters. Therefore, the better we understand the behavior of the parameters, the more accurate our analysis will be, and we can guarantee that the evidence found will represent the curve with the best fit [48]. In Figure 5, Figure 6 and Figure 7, we have scatter plots for the parameters of the models (9) (a) and (11) (b). For all chromosomes of all species analyzed here, we found strong correlations between the parameters $\gamma_{1}$ and $\gamma_{2}$ present in the distribution (9). This was expected, as this model appears as a variation of the model (6), as carried out in [34]. These two adjustable constants together ($\gamma_{1}$ and $\gamma_{2}$) have an inverse role to what $L_{\kappa }$ has in the distribution (6), and when $\gamma_{1}=\gamma_{2}$, we obtain the model (6) again. This implies that these parameters are related to the κ parameter in the same way, resulting in similar images for scattering but with different ranges. This behavior was repeated for all chromosomes.

**Figure 8:**
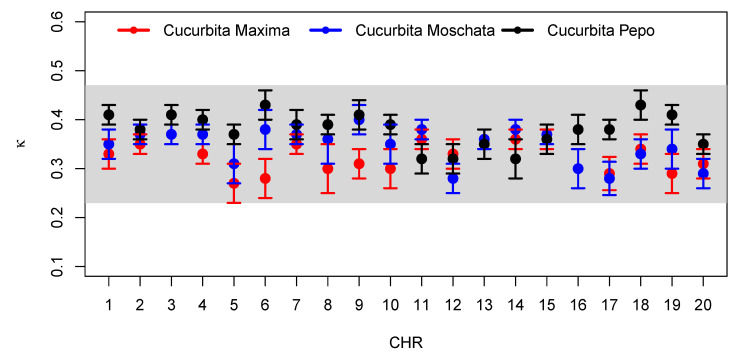
κ values, from the best fit model, Equation (9), for different species. In red, blue, and black, we have, respectively, *Cucurbita maxima*, *Cucurbita moschata*, and *Cucurbita pepo*.

The $\kappa_{S}$ parameter (that is, the κ value that provides the best fit, when using the sum of κ-exponentials, Equation (9)) in Table A4, Table A5 and Table A6, measures the correlation between lengths *l*, and belongs to the range (0.27(4);0.37(2)) in the case of *Cucubita maxima*, (0.28(3);0.40(4)) for *Cucubita moschata*, and (0.32(3);0.43(3)) for *Cucubita pepo*. It can be seen in Figure 8 that the values of κ, for different species, seem to specify a universal behavior. Therefore, all of these findings lead us to the conclusion that for all the species under study, the model (9) (sum of κ-exponentials) is strongly preferred over the distribution model (11) (κ-Maxwellian).

## 4. Conclusions

A statistical model based on non-additive statistics was developed to describe the size distribution of nucleotide chains in the DNA of species belonging to the *Cucurbitaceae* family, namely *Cucurbita maxima*, *Cucurbita moschata*, and *Cucurbita pepo* [26,27,28,31]. Specifically, the proposed distribution, Equation (9), expands on a distribution studied in [41] through the sum of the κ-exponentials, which added the parameters $\gamma_{1}$ and $\gamma_{2}$ to capture the statistical correlations between the DNA strands. Another model investigated was the κ-Maxwellian distribution, Equation (11), proposed in [31] for human DNA. We tested the statistical feasibility of models, as well as methods based on Bayesian statistical analysis using the NCBI project database. The cumulative distribution function (9) best fitted the nucleotide base for all chromosomes, of the three species, with the parameter κ belonging to the range (0.27(4);0.37(2)) for *Cucurbita maxima*, (0.28(3);0.40(4)) for *Cucurbita moschata*, and (0.32(3);0.43(3)) in the case of *Cucurbita pepo*. It can be seen in Figure 8 that the values of κ for different species of the coding parts (exons) of the DNA appear to be within a common and relatively narrow range.

Regarding the Bayesian analysis, we compared the κ-exponential-sum distribution with the κ-Maxwellian model. We demonstrated that the first has solid and favorable evidence compared to the κ-Maxwellian distribution. This was reasonably expected given that the distribution (9) has a free parameter for potential future adjustments. A general task should be to expand the model presented in this study to include additional species, determining whether they fall within the same range of κ for exons (0.35±0.08) discovered for the species investigated here.

## Data Availability

The DNA code data that support the findings of this study are available in NCBI [42].

## Appendix A

**Table A1:** Statistical characteristics of the data after outliers are removed. The first column indicates the chromosome, the second the number of exons, the third and fourth the minimum and maximum lengths. Finally, the quartiles Q1, Q2, and Q3 are in the fifth, sixth, and seventh columns, respectively. In the later columns, we have indicated the same parameters for the other species of *cucurbitaceae*.

CHR	*Cucurbita maxima*							*Cucurbita moschata*							*Cucurbita pepo*					
	*N*	$l_{min}$	$l_{max}$	Q1	Q2	Q3		*N*	$l_{min}$	$l_{max}$	Q1	Q2	Q3		*N*	$l_{min}$	$l_{max}$	Q1	Q2	Q3
1	662	1	2034	166	350	738		667	1	1965	168	357	737		802	3	2049	208	451	833
2	666	1	2229	168	362	710		653	1	2169	165	363	741		663	3	1854	173	380	755
3	641	1	2316	162	352	744		610	1	2207	155	333	740		661	4	1804	172	381	721
4	790	1	2049	198	424	815		851	1	2070	214	458	871		572	4	2004	159	350	687
5	596	1	1967	151	321	650		605	1	1997	154	324	670		623	3	1953	162	352	685
6	654	1	1981	164	343	677		657	1	2147	165	369	747		503	4	1899	146	313	635
7	568	1	2127	143	309	692		608	1	2148	153	343	771		538	3	1917	145	315	650
8	534	2	1857	135	296	587		513	1	1932	130	284	609		578	4	1815	151	339	631
9	610	1	2361	153	320	647		599	1	2363	154	38	753		539	3	1866	152	330	647
10	532	1	2096	134	285	598		616	1	2346	157	342	701		529	4	1995	146	330	703
11	665	1	2001	167	353	753		714	1	2146	179	399	803		588	3	1773	158	337	618
12	557	1	2265	141	302	622		556	1	2133	141	293	610		570	3	1776	150	329	602
13	549	3	2037	141	307	675		605	1	2136	155	338	750		563	3	2092	153	329	606
14	723	1	2181	182	391	786		740	3	2382	188	410	832		534	3	1896	143	311	589
15	601	1	2121	152	325	675		630	1	2190	159	338	673		486	4	1896	137	321	603
16	597	1	2153	150	318	657		601	1	2188	155	333	660		512	3	1872	137	302	649
17	583	2	2052	148	312	614		641	2	1968	164	341	672		507	3	1782	140	333	695
18	574	1	2178	145	320	635		580	1	2016	146	317	624		445	4	2052	129	313	611
19	539	1	2124	137	301	648		555	1	2049	141	310	641		514	3	1884	151	347	697
20	564	1	2212	142	298	628		572	1	2427	145	300	683		501	4	1914	143	316	650

**Table A2:** Values of κ before and after removing outliers for model (9). (*N*) represents the number of nucleotides. κ are the best fit values. The subscripts 0 and *f* represent the values before and after the outliers are removed, (RD) represents the relative difference between them. This behavior is repeated for all chromosomes.

CHR	*Cucurbita maxima*						*Cucurbita moschata*						*Cucurbita pepo*				
	$N_{0}$	$\kappa_{0}$	$N_{f}$	$\kappa_{f}$	|RD|		$N_{0}$	$\kappa_{0}$	$N_{f}$	$\kappa_{f}$	|RD|		$N_{0}$	$\kappa_{0}$	$N_{f}$	$\kappa_{f}$	|RD|
1	687	0.3324	662	0.3336	0.0012		693	0.3455	667	0.3455	0.0000		834	0.4061	802	0.4073	0.0012
2	688	0.3532	666	0.3546	0.0014		675	0.3742	653	0.3749	0.0007		683	0.3812	663	0.3836	0.0024
3	661	0.3749	641	0.3747	0.0002		630	0.3641	610	0.3650	0.0009		682	0.4057	661	0.4076	0.0019
4	828	0.3576	790	0.3330	0.0246		893	0.3677	851	0.3680	0.0003		587	0.4004	572	0.4015	0.0011
5	615	0.2827	596	0.2835	0.0008		623	0.3127	605	0.3123	0.0004		643	0.3661	623	0.3673	0.0012

**Table A3:** The same as Table A2, but for model (11).

CHR	*Cucurbita maxima*						*Cucurbita moschata*						*Cucurbita pepo*				
	$N_{0}$	$\kappa_{0}$	$N_{f}$	$\kappa_{f}$	|RD|		$N_{0}$	$\kappa_{0}$	$N_{f}$	$\kappa_{f}$	|RD|		$N_{0}$	$\kappa_{0}$	$N_{f}$	$\kappa_{f}$	|RD|
1	687	0.6589	662	0.6590	0.0002		693	0.6648	667	0.6649	0.0001		834	0.6929	802	0.6930	0.0001
2	688	0.6704	666	0.6707	0.0003		675	0.6810	653	0.6811	0.0001		683	0.6919	663	0.6922	0.0003
3	661	0.6760	641	0.6762	0.0000		630	0.6733	610	0.6734	0.0001		682	0.6909	661	0.6911	0.0002
4	828	0.6588	790	0.6592	0.0096		893	0.6633	851	0.6633	0.0000		587	0.7067	572	0.7071	0.0004
5	615	0.6455	596	0.6459	0.0002		623	0.6555	605	0.6556	0.0001		643	0.6881	623	0.6885	0.0004

**Table A4:** The average of the best fit parameters for the *Cucurbita maxima* species. The sub-index *S* and *M* represent the κ-exponential sum function (9) and the κ-Maxwellian function (11), respectively. $\sigma_{\kappa }$, $\gamma_{1}$, and $\gamma_{2}$ are free parameters related to the length of the nucleotide chain. The numbers in parenthesis denote the calculated errors.

CHR	*N*	$\kappa_{M}$	$\sigma_{\kappa }$	$\kappa_{S}$	$\gamma_{1}$	$\gamma_{2}$
1	662	0.65(1)	97(2)	0.33(3)	0.0067(13)	0.0057(07)
2	666	0.67(1)	101(3)	0.35(2)	0.0054(09)	0.0062(14)
3	641	0.67(1)	95(3)	0.37(2)	0.0069(15)	0.0057(08)
4	790	0.65(1)	94(2)	0.33(2)	0.0067(13)	0.0056(08)
5	596	0.64(1)	102(3)	0.27(4)	0.0054(13)	0.0062(18)
6	654	0.64(1)	96(2)	0.28(4)	0.0056(08)	0.0069(15)
7	568	0.67(1)	92(3)	0.35(2)	0.0059(09)	0.0071(17)
8	534	0.65(1)	94(3)	0.30(5)	0.0060(18)	0.0066(23)
9	610	0.66(1)	103(2)	0.31(3)	0.0051(07)	0.0061(12)
10	532	0.66(1)	99(3)	0.30(4)	0.0053(08)	0.0066(16)
11	665	0.67(1)	96(3)	0.36(2)	0.0069(14)	0.0057(08)
12	557	0.66(1)	90(3)	0.33(3)	0.0059(09)	0.0072(16)
13	549	0.67(1)	95(3)	0.36(2)	0.0067(15)	0.0055(08)
14	723	0.66(1)	96(2)	0.36(2)	0.0068(13)	0.0057(07)
15	601	0.68(1)	91(2)	0.36(2)	0.0068(13)	0.0058(08)
16	597	0.65(1)	98(3)	0.30(4)	0.0067(16)	0.0055(08)
17	583	0.65(1)	102(3)	0.29(4)	0.0051(07)	0.0062(14)
18	574	0.67(1)	93(3)	0.34(3)	0.0070(17)	0.0057(09)
19	539	0.65(1)	98(3)	0.29(4)	0.0065(14)	0.0053(08)
20	564	0.66(1)	101(3)	0.31(3)	0.0052(08)	0.0063(13)

**Table A5:** The same as Table A4, but for the *Cucurbita moschata* species. The numbers in parenthesis denote the calculated errors.

CHR	*N*	$\kappa_{M}$	$\sigma_{\kappa }$	$\kappa_{S}$	$\gamma_{1}$	$\gamma_{2}$
1	667	0.66(1)	95(2)	0.35(3)	0.0069(14)	0.0057(08)
2	653	0.68(1)	97(2)	0.37(3)	0.0054(07)	0.0066(14)
3	610	0.67(1)	96(2)	0.37(3)	0.0068(14)	0.0056(08)
4	851	0.66(1)	96(2)	0.37(3)	0.0071(13)	0.0059(07)
5	605	0.65(1)	103(3)	0.31(2)	0.0052(08)	0.0063(14)
6	657	0.68(1)	97(2)	0.38(4)	0.0065(14)	0.0055(08)
7	608	0.67(1)	92(3)	0.37(3)	0.0074(19)	0.0059(09)
8	513	0.67(1)	88(3)	0.36(2)	0.0074(18)	0.0060(09)
9	599	0.69(1)	96(2)	0.40(4)	0.0054(07)	0.0064(12)
10	616	0.66(1)	98(3)	0.35(3)	0.0068(17)	0.0055(08)
11	714	0.68(1)	99(2)	0.38(2)	0.0054(06)	0.0064(11)
12	556	0.64(1)	99(3)	0.28(3)	0.0054(09)	0.0066(17)
13	605	0.67(1)	104(3)	0.36(3)	0.0051(07)	0.0062(14)
14	740	0.67(1)	95(2)	0.38(3)	0.0059(08)	0.0070(14)
15	630	0.67(1)	90(2)	0.37(4)	0.0070(13)	0.0059(07)
16	601	0.65(1)	107(3)	0.30(3)	0.0049(07)	0.0058(12)
17	641	0.64(1)	105(3)	0.28(3)	0.0061(13)	0.0051(08)
18	580	0.66(1)	99(3)	0.33(4)	0.0064(14)	0.0052(07)
19	555	0.67(1)	99(2)	0.34(3)	0.0051(07)	0.0062(12)
20	572	0.65(1)	110(2)	0.29(2)	0.0047(07)	0.0057(12)

**Table A6:** The same as Table A4, but for the *Cucurbita pepo* species. The numbers in parenthesis denote the calculated errors.

CHR	*N*	$\kappa_{M}$	$\sigma_{\kappa }$	$\kappa_{S}$	$\gamma_{1}$	$\gamma_{2}$
1	802	0.69(1)	88(2)	0.41(2)	0.0072(16)	0.0059(08)
2	663	0.69(1)	97(3)	0.38(2)	0.0053(07)	0.0062(12)
3	661	0.69(1)	84(3)	0.41(2)	0.0064(09)	0.0077(17)
4	572	0.70(1)	95(2)	0.40(2)	0.0062(14)	0.0051(07)
5	623	0.68(1)	96(3)	0.37(2)	0.0061(13)	0.0053(09)
6	503	0.70(1)	75(2)	0.43(3)	0.0070(13)	0.0091(31)
7	538	0.69(1)	87(3)	0.39(3)	0.0059(09)	0.0074(20)
8	578	0.70(1)	94(3)	0.39(2)	0.0061(12)	0.0052(08)
9	539	0.69(1)	77(2)	0.41(3)	0.0088(29)	0.0068(12)
10	529	0.70(1)	94(3)	0.39(2)	0.0063(14)	0.0051(08)
11	588	0.67(1)	109(3)	0.32(3)	0.0045(06)	0.0053(09)
12	570	0.67(1)	96(3)	0.32(3)	0.0063(16)	0.0053(10)
13	563	0.69(1)	92(3)	0.35(3)	0.0053(08)	0.0064(13)
14	534	0.67(1)	95(2)	0.32(4)	0.0053(09)	0.0067(17)
15	486	0.69(1)	88(2)	0.36(3)	0.0066(17)	0.0053(08)
16	512	0.69(1)	90(3)	0.38(3)	0.0055(08)	0.0067(16)
17	507	0.69(1)	94(3)	0.38(2)	0.0065(14)	0.0053(07)
18	445	0.71(1)	77(3)	0.43(3)	0.0074(27)	0.0065(12)
19	514	0.71(1)	93(3)	0.41(2)	0.0065(14)	0.0051(07)
20	501	0.69(1)	106(3)	0.35(2)	0.0045(06)	0.0053(09)

**Table A7:** Bayesian analysis for exons of each chromosome. The column ln(E) gives us the Baysian evidence for each of the models, Equation (11) for (i) and (9) for (j). The indices *max*, *mos*, and *pep* represent, respectively, the species *Cucurbita maxima*, *Cucurbita moschata*, and *Cucubita pepo*. The numbers in parenthesis indicate the calculated errors.

CHR	κ-Maxwellian			Sum κ-Exponentials			Bayes Factor		
	$\ln(E_{i}^{\max})$	$\ln(E_{i}^{\mathrm{mos}})$	$\ln(E_{i}^{\mathrm{pep}})$	$\ln(E_{j}^{\max})$	$\ln(E_{j}^{\mathrm{mos}})$	$\ln(E_{j}^{\mathrm{pep}})$	$\ln(B_{\mathrm{ij}}^{\max})$	$\ln(B_{\mathrm{ij}}^{\mathrm{mos}})$	$\ln(B_{\mathrm{ij}}^{\mathrm{pep}})$
1	−147.17(1)	−142.12(1)	−170.98(1)	−135.34(1)	−128.84(2)	−152.95(6)	−11.83(1)	−13.28(2)	−18.03(6)
2	−144.46(1)	−137.10(1)	−130.95(1)	−133.29(3)	−125.46(6)	−117.92(1)	−11.17(3)	−11.64(6)	−13.03(1)
3	−138.97(1)	−127.36(1)	−133.76(1)	−128.22(3)	−116.33(1)	−118.96(6)	−10.75(3)	−11.03(1)	−14.80(6)
4	−187.07(1)	−197.18(1)	−107.60(1)	−172.44(7)	−180.54(1)	−97.81(1)	−14.63(7)	−16.64(1)	−9.79 (1)
5	−128.49(1)	−126.69(1)	−121.06(1)	−117.14(3)	−115.10(4)	−108.52(2)	−11.35(3)	−11.59(4)	−12.54(2)
6	−146.70(1)	−137.90(1)	−94.60 (1)	−133.68(2)	−126.53(1)	−83.70(5)	−13.02(2)	−11.37(1)	−10.90(5)
7	−120.97(1)	−131.00(1)	−104.27(1)	−111.31(3)	−119.85(2)	−93.34(3)	−9.66 (3)	−11.15(2)	−10.93(3)
8	−112.37(1)	−103.10(1)	−105.71(1)	−102.26(2)	−93.35(2)	−94.89(2)	−10.11(2)	−9.75 (2)	−10.82(2)
9	−129.29(1)	−120.78(1)	−104.51(1)	−119.07(2)	−111.31(3)	−92.33(1)	−10.22(2)	−9.47 (3)	−12.18(1)
10	−110.92(1)	−132.10(1)	−100.74(1)	−101.87(1)	−120.77(2)	−91.92(3)	−9.05 (1)	−11.33(2)	−8.82 (3)
11	−146.58(1)	−152.99(1)	−108.48(1)	−135.32(1)	−140.62(1)	−97.11(3)	−11.26(1)	−12.37(1)	−11.37(3)
12	−116.96(1)	−114.18(1)	−110.76(1)	−106.53(3)	−102.74(2)	−98.23(6)	−10.43(3)	−11.44(2)	−12.53(6)
13	−114.54(1)	−125.34(1)	−105.07(1)	−105.62(2)	−115.35(3)	−93.44(3)	−8.92 (2)	−9.99 (3)	−11.63(3)
14	−163.94(1)	−163.55(1)	−103.71(1)	−151.50(4)	−150.13(3)	−92.43(1)	−12.44(4)	−13.42(3)	−11.28(1)
15	−126.73(1)	−129.25(1)	−90.10 (1)	−116.28(1)	−116.91(2)	−80.08(2)	−10.45(1)	−12.34(2)	−10.02(2)
16	−130.03(1)	−124.87(1)	−96.78 (1)	−119.19(3)	−114.18(1)	−87.58(1)	−10.84(3)	−10.69(1)	−9.20 (1)
17	−121.57(1)	−135.30(1)	−98.24 (1)	−111.25(2)	−122.31(5)	−89.39(1)	−10.32(2)	−12.99(5)	−8.85 (1)
18	−121.37(1)	−117.42(1)	−84.51 (1)	−111.18(2)	−106.33(1)	−76.01(2)	−10.19(2)	−11.09(1)	−8.50 (2)
19	−114.09(1)	−110.97(1)	−95.81 (1)	−104.59(1)	−101.15(1)	−87.73(1)	−9.50 (1)	−9.82 (1)	−8.08 (1)
20	−117.37(1)	−116.36(1)	−91.77 (1)	−108.02(1)	−106.56(2)	−83.96(1)	−9.35 (1)	−9.80 (2)	−7.81 (1)
